# *Plasmodium berghei* ANKA Infection in ICR Mice as a Model of Cerebral Malaria

**Published:** 2012

**Authors:** R Basir, SS Fazalul Rahiman, K Hasballah, WC Chong, H Talib, MF Yam, M Jabbarzare, TH Tie, F Othman, MAM Moklas, WO Abdullah, Z Ahmad

**Affiliations:** 1 Department of Human Anatomy, Faculty of Medicine & Health Sciences, Universiti Putra Malaysia, 43400 Serdang, Selangor, Malaysia; 2 Department of Pharmacology, Faculty of Medicine, Syiah Kuala University, 23111 Darussalam, Banda Aceh, Nanggroe Aceh Darussalam, Indonesia; 3 Department of Pathology, Faculty of Medicine & Health Sciences, Universiti Putra Malaysia, 43400 Serdang, Selangor, Malaysia; 4 Pharmacology Discipline, School of Pharmaceutical Sciences, Universiti Sains Malaysia, 11800 Penang, Malaysia; 5 Department of Medical Microbiology and Parasitology, Faculty of Medicine & Health Sciences, Universiti Putra Malaysia, 43400 Serdang, Selangor, Malaysia

**Keywords:** Animal model, Malaria, *Plasmodium berghei*, ICR mice

## Abstract

**Background:**

Animal models with various combination of host-parasite have long been employed to study malaria pathogenesis. Here, we describe the combination of *Plasmodium berghei* ANKA infection in inbred ICR mice as a model of cerebral malaria (CM).

**Methods:**

Infection in mice was initiated by intraperitoneal injection of 2 x 10^7^ (0.2ml) parasitized red blood cells (PRBCs).

**Results:**

This model can produce a severe degree of infection presented by the high degree of parasitaemia followed by death 6-7 days post infection. Severe anemia, splenomegaly, hepatomegaly and discolourations of major organs were observed. Histopathological findings revealed several important features mimicking human CM including, microvascular sequestration of PRBCs in major organs, particularly in the brain, hypertrophy and hyperplasia of the kupffer cells in the liver, pulmonary edema and hyaline membrane formation in the lungs and haemorrhages in the kidney's medulla and cortex. Proinflammatory cytokines TNFα, IFNγ, IL-1, IL-6 and IL-18, and anti-inflammatory cytokine IL-10 were all found to be elevated in the plasma of infected mice.

**Conclusion:**

This model can reproduce many of the important features of CM and therefore can be used as a tool to advance our understanding of the disease pathogenesis.

## Introduction

Experimental animal models have been paramount towards the understanding of human diseases. They represent the first crucial step in the research of the underlying disease pathogenesis and development of new drug compounds and vaccines (1), and had paved the way into a modern biomedical research without which we would know very little. Animals are employed as models of human diseases mainly due to the impossibility of some research procedures to be carried out in humans for practical or ethical reasons. Despite the limitations in mimicking every features of the human disease, majority of the current available knowledge basically relies on animal model data. Many approaches to understanding pathogenesis of disease have involved experimental animal model and *in vitro* modelling of the pathogenic process. New experimental animal models should be considered as an additional strength as they will undoubtedly bring advances in our knowledge and understanding of human pathological conditions, but their relevance must be precisely validated.

Malaria is undoubtedly still considered as the most serious tropical disease that afflicts mankind throughout the world with the greatest impact in the sub-saharan Africa. During 2010, a total of 106 countries worldwide were considered as endemic of malaria with an estimated 3.3 billion people were at risk of the disease. The disease burden in 2010 was estimated at 216 million cases with 655,000 estimated deaths worldwide, in which, 86% were in children under 5 years of age (2). The disease is caused by protozoan parasite from the genus *Plasmodium (P.)* with female *Anopheles* mosquito acted as the vector. Four species of *Plasmodium* are known to infect human, namely *P. Ovale, P. Vivax, P. Malariae* and *P. falciparum*, with the latest being the most virulent and lethal.

So far, malarial models have been developed in monkey, rats and mice, and although they cannot exactly mimic the full human syndrome of the disease, they do faithfully represent certain aspects of human malaria. The mouse model has been extensively studied due to the numerous similarities between the defined malaria antigens in murine and human parasites and also between the murine and human immune response pathway. The many species and strains of *Plasmodium* available and the very large selection of inbred and outbreed laboratory rodents has created a great number of mouse-parasite combinations that can be used experimentally. The most widely used model of malaria so far is *P. berghei* ANKA infection in CBA or C57BL/6 mice (3, 4).

In this study, we investigated the validity of using *P. berghei* ANKA infection in ICR mice as a model of severe malaria infection. *Plasmodium berghei* ANKA strain is the parasite of choice because of its ability to sequester within the microcirculation which is the characteristic of severe malaria especially the cerebral form (5).

Cerebral malaria (CM), the major severe form of the infection, is lethal and mainly resulted from *P. falciparum* infection. It is characterized by a sequestration of parasitized red blood cells (PRBC), particularly in the deep cerebral vascular beds and by elevated levels of pro-inflammatory cytokines such as TNFα, IL-1, IL-6, IFNγ etc. (6, 7). The fine mechanisms leading to cerebral complications in human CM remains incompletely understood and the understanding of the pathogenesis of CM necessarily relies on the use of experimental animals or *in vitro* models (8). Analysis of cerebral pathology in human patients is almost impossible and the full extent of the pathogenic events leading to severe complications in *falciparum* infection remains largely unresolved since the outcome from post-mortem investigation can only provide the endpoint findings. The existing animal models of CM need to be improved and their advantages and disadvantages must be systematically evaluated. New model systems with new host-pathogen combination and well defined parameters will also need to be developed.

It is hopeful that the introduction of a new model of malaria in this study with new combination of mouse-parasite and well-defined pathophysiological features that mimic the human disease, will pave a way to a better understanding of the underlying processes leading to the severe manifestation and death in severe malaria cases, especially CM.

Here, we describe the combination of *P. berghei* ANKA infection in inbred ICR mice as a model of cerebral malaria (CM).

## Materials and Methods

### Animals

This study was conducted at Universiti Putra Malaysia from January 2011 till December 2011. Male ICR mice weighing initially between 18-20g were used in this study. The mice were obtained from the Faculty of Medicine & Health Sciences Animals Unit, Universiti Putra Malaysia where they were maintained at a constant room temperature (25-27 °C) on a 12 hour light (0800-2000) and dark (2000-0800) cycle with unlimited access to food (CRM feeding pellets) and water. Animals were handled as gently as possible and transferred from the Unit to the laboratory at least 30-60 min prior to use, in order to minimise the effects of stress. All procedures conducted on the animals were in accordance to the rules and regulations set by the Animals Care and Use Committee (ACUC) of Universiti Putra Malaysia (Ethical approval number: UPM/FPSK/PADS/BR-UUH/00365).

### Parasite and infection procedures

The rodent malaria parasite, *P. berghei ANKA* strain (chloroquine sensitive), was originally obtained from The Institute of Medical Research, Kuala Lumpur, Malaysia. The parasite has been maintained at Universiti Putra Malaysia by combination of passage in male ICR mice and cryoscopic storage. Serial passage of *P. berghei* was initiated after storage at -70 °C by intraperitoneal (i.p) injection into normal mice of 0.2ml blood containing parasites stored in Alservers buffer solution and allowed for 3 or 4 days depending on the development of the parasites in the mice. Subsequent passage for maintaining the parasites were carried out by i.p inoculation of normal mouse with 0.2 ml blood, diluted to contain 2 × 10^7^ parasitized red blood cells (PRBC) from a donor mouse. Passage was repeated every three or four days (with parasitaemia levels reaching at least 35%). Parasitaemia was measured on the day of passage and 0.1ml minimum of blood was removed from the infected mouse by cardiac puncture while the animal was under general anesthesia induced by inhalation of diethyl ether. Blood was diluted with sterile, non-pyrogenic 0.85% saline to give 2 × 10^7^ PRBC in an injection volume of 0.2ml and the percentage parasitaemia determined the amount of saline used in the dilution of the infected blood (e.g., if 1ml of blood was removed then dilution was with (x-1)ml of saline, where x was the percentage parasitaemia). Infection in the experimental mice was carried out by injecting 0.2 ml of 2 × 10^7^ PRBC (i.p). Control to the malarial mice received an equivalent volume and dilution of normal uninfected red blood cells.

### Measurements of basic parameters

The body weight of mice was measured throughout the study using a Shimadzu (UX4200H) top pan animal balance to the nearest 0.1g. Colonic temperature in mice was measured using a BIOSEB (BIO9882) rectal thermometer with a probe inserted approximately 1.5 cm past the anal sphincter into the colon of hand-held mice. All control and malaria-infected mice were observed visually throughout the experiment for behavioural changes and signs of illness which include diarrhoea, lethargy, piloerection, and decreased locomotor activity. Any signs of illness were quantified using arbitrary scale and recorded as either absent (–), mild (+), moderate (+ + ) or severe (+ + +). Infection in the mice was allowed to continue until all the infected mice died of the infection. Mortality was recorded throughout the experiment. *Post mortem* examinations were carried out for any observable changes in the appearance of the liver, spleen and brain of both control and infected mice.

### Parasitaemia measurement

A drop of blood was collected from the mice by venesection of the tail and transferred onto the edge of a microscope slide (single, 76 × 26 mm thickness). The blood was drawn evenly across a second slide to make a thin blood film and allowed to dry at room temperature before staining with Leishman stain. Slides were viewed using light microscopy (Vickers Instruments) with oil immersion (1000x magnification). Parasitaemia was counted based on the Leishman positive bodies which represent the parasitized red blood cells. The Leishman positive cells were counted with the aid of a graticule and hand counter. Five fields of approximately 200 cells each were counted and the parasitaemia was calculated as the percentage of the total red blood cells containing Leishman positive bodies.

### Cytokines measurement in the plasma

Five major cytokines were measured in the plasma of control and malarial mice which include the pro-inflammatory cytokines TNFα, IFNγ, IL-1, IL-18 and IL-6 and the anti-inflammatory cytokine IL-10. All the cytokines were measured by means of ELISA method using the commercially available kits (R&D System, USA). The assay was carried out according to the manufacturer's procedures and protocols. Blood samples for plasma were collected from the mice by cardiac puncture while they were terminally anaesthetised by inhalation of diethyl ether. Blood was taken using a sterile disposable syringe containing 0.05ml heparin (1000U/ml) and transferred into a sterile non-pyrogenic glass test tube before centrifugation (MSE, Centaur 2) at 2500 rpm for 10 min to separate the plasma from the blood cells.

### Histopathological study

Five major organs including the brain, liver, lungs, spleen and kidneys were collected from the animals on day 5 post-infection and preserved in 10% of buffered formalin for histopathological study. Organ collections were carried out on day 5 post infection to avoid losing the fresh organs due to the high mortality rate of infected mice on day 6. The organ specimens were subjected to a tissue processing cycle using an automated tissue processor (Leica, Germany) to remove the water from the tissues. Following that, the specimens were embedded into melted paraffin wax using a histoembedder (Leica, Germany) and sectioned into a 4.0µm thick slice with a microtome (Leica, Germany). Tissue sections were then stained with hematoxylin and eosin (H&E) using an autostainer (Leica, Germany). The morphological changes within the tissues of the control and infected mice were observed under light microscopy with 100x, 200x and 400x magnifications.

### Statistical analysis

Results were analysed statistically using Graph Pad Prism software and SPSS version 16. Group means from the experiment were compared using one-way ANOVA (analysis of variance) followed by Tukey's test as a single post-hoc test. All results are expressed as mean ± s.e.m (standard error of the means). *P*<0.05 is taken as statistically significant.

## Results

### Parasitaemia development

**Fig. 1:**
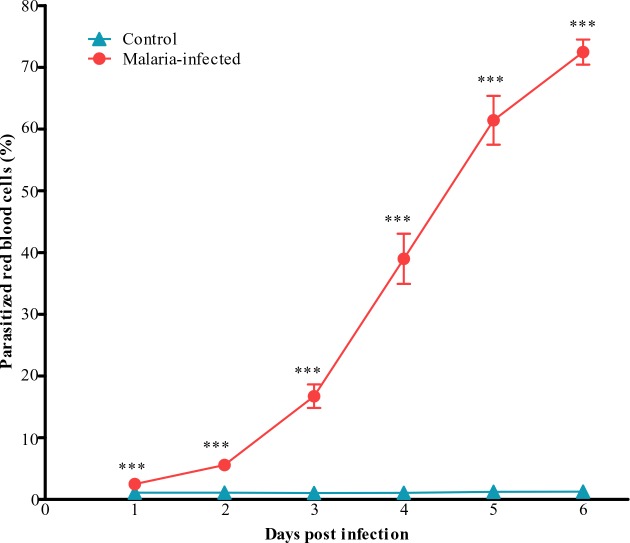
Parasitaemia measured in the control and malaria-infected mice. Parasitized red blood cells were taken as an index of parasitaemia. Results are mean ± s. e. m with N = 8, ***P<0.0005

Parasitaemia levels were taken as the percentage of parasitized red blood cells measured. In the malarial mice, an increase in parasitaemia with increasing days after inoculation were observed with peak parasitaemia occurred on day 6 (Fig. 1).

**Fig. 2:**
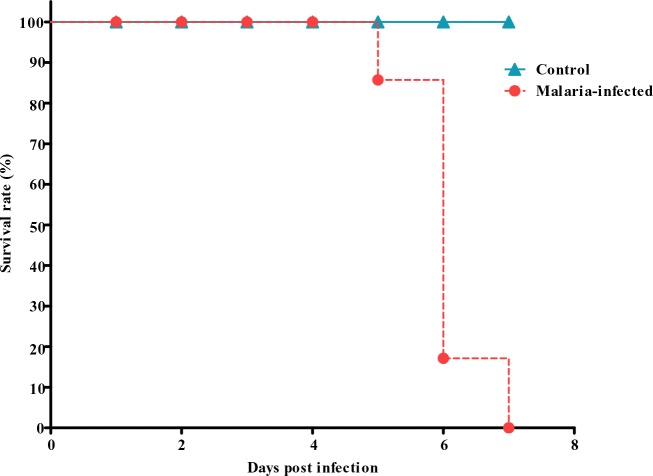
Percentage survival of control and malaria infected mice following inoculation with the parasites

Control mice on all days showed Leishman positive cells between 1-2% and served as the baseline. A high degree of parasitaemia was observed in this species, peaking on day 6 (60-80%). This model of malaria infection is lethal and death would follow the peak parasitaemia. 20% mortality was recorded on day 5 post infection, followed by 70% mortality on day 6 and by day 7, 100% mortality were observed (Fig. 2).

**Fig. 3:**
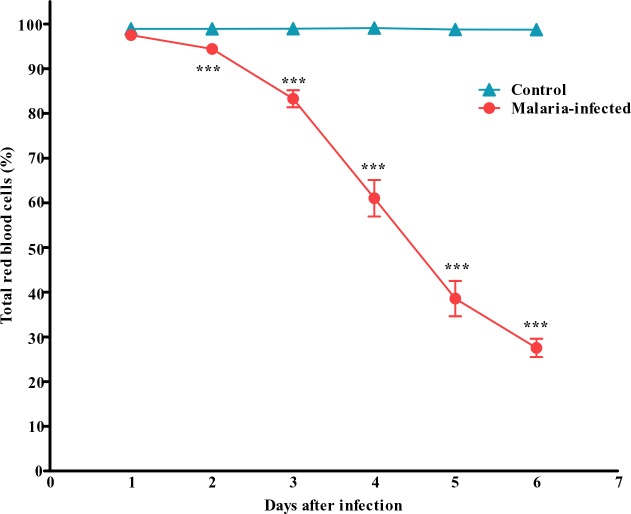
Total red blood cells counted in the control and malaria-infected mice. Results are mean ± s.e.m of N = 8. ***P<0.0005

Total count on the normal red blood cells (RBCs) also showed a tremendous decrease in the number with more than 70% reduction in the total number of RBC during late critical phase of the infection (Fig. 3).

### Basic parameters and visual observations

**Fig. 4:**
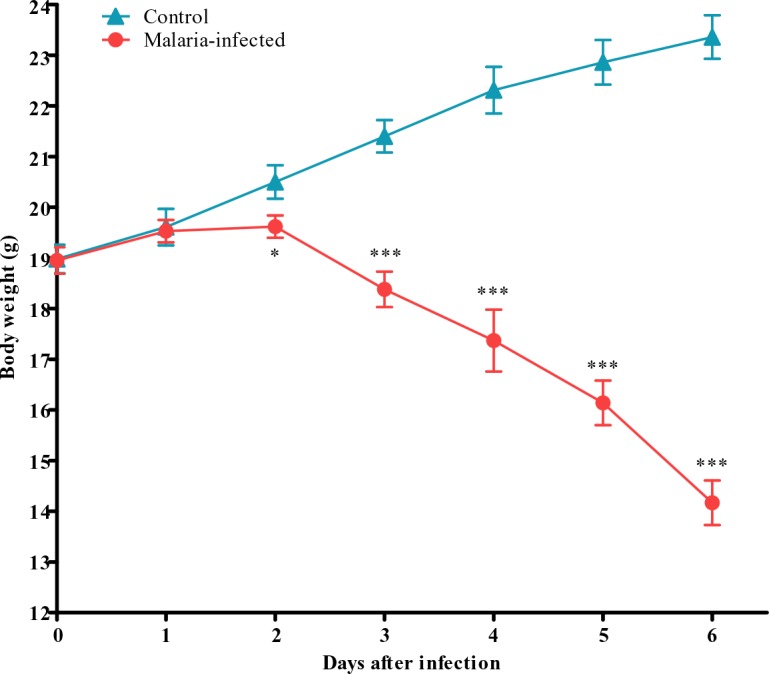
Body weight of control and malaria-infected mice. Results are mean ± s.e.m of N = 8, *P<0.05, ***P<0.0005

The overall mean values of body weight in the malaria-infected mice were significantly decreased as compared to the control uninfected group (Fig. 4).

**Fig. 5:**
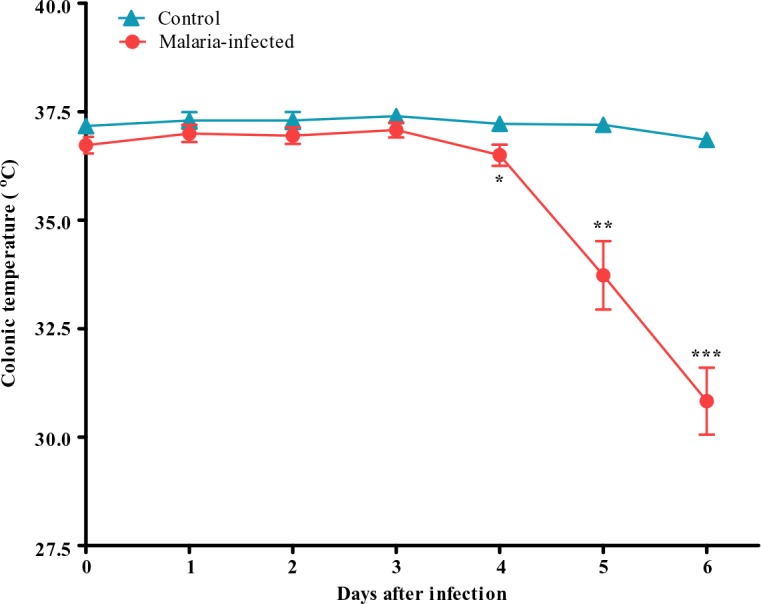
Colonic temperature of control and malaria-infected mice. Results are presented as the mean ± s.e.m of N = 8, *P<0.05, **P<0.005, ***P<0.0005

Control group progressively gain weight until the end of the experiment on day 6 whereas a progressive decrease in body weight was observed in malarial mice. The average weight gain in the control group was 0.73 ± 0.37g/day whereas malarial infected group showed an average weight loss of 0.79 ± 0.36g/day. The body temperature changes in both control and malaria-infected mice measured as colonic temperature are shown in Fig. 5.

Significant changes on body temperature in malaria-infected mice were observed starting on day 4 following infection. A gradual decrease in body temperature was observed with body temperature fall as low as 32 °C on day 6 following infection (Fig. 5).

Post mortem examinations on the internal organs revealed the darkening of the liver, spleen, lungs and the kidneys of the malaria-infected mice (Table 1).

**Table 1:** Post mortem observations on five major organs in malaria-infected mice

Organ	Post-mortem observations
Spleen	Enlargement of the size (splenomegaly);
Discolouration of the spleen	
Liver	Enlargement of the size (hepatomegaly);
Discolouration of the liver	
Brain	Paleness of the brain
Kidneys	Discolouration of the kidneys
Lungs	Discolouration of the lungs

Paleness of the brain was also observed in the infected group as compared to the control. Malarial mice also showed enlarged spleen and liver indicating splenomegaly and hepatomegaly respectively. The wet weights of all the organs recorded a gradually increased towards the late stages of the infection as compared to the controls (Table 2). Physical signs of illness including piloerection, lethargy, decreased locomotor activity and passage of dark/black urine were observed during the late critical stages of the infection (Table 3).

**Table 2:** Wet weight of five major organs of control and malaria-infected mice

Organ	Control (mg)	Malaria-infected mice (mg)					
		Day 1	Day 2	Day 3	Day 4	Day 5	Day 6
Liver	843±6.66	854.11±4.29	1184.50±8.45	1384.67±15.24	1451.33±13.66	1562.72±4.67	1633.89±6.25
Spleen	81.56±2.66	88.83±1.85	129.28±0.96	150.89±14.73	156.89±2.03	177.06±3.12	202.56±2.09
Brain	277.44±4.92	312.50±4.57	334.17±4.56	348.06±5.39	360.33±16.04	360.44±15.69	366.17±1.48
Lungs	150.44±2.38	176.06±3.24	180.56±2.69	192.39±3.28	208.33±4.71	236.00±6.68	253.61±11.53
Kidneys	225.00±5.55	259.00±5.37	278.78±2.68	283.61±0.67	294.39±12.55	305.17±10.41	331.06±4.91

**Table 3:** Physical signs of illness in control and malaria-infected mice

Illness behaviours	Days after inoculation	Control	Malaria-infected
	1	−	−
	2	−	−
**Piloerection**	3	−	-
	4	−	+
	5	−	+ +
	6	−	+ + +
	1	−	−
	2	−	−
**Lethargy**	3	−	−
	4	−	+
	5	−	+ +
	6	−	+ + +
	1	−	−
	2	−	−
**Reduction in locomotor activity**	3	−	−
	4	−	+
	5	−	+ +
	6	−	+ + +
	1	−	−
	2	−	−
**Passage of dark urine**	3	−	−
	4	−	−
	5	−	+ +
	6	−	+ + +

Indicator: (−) absent; (+): mild; (+ + ): moderate; (+ + +): severe

### Cytokines levels in the plasma

**Fig. 6:**
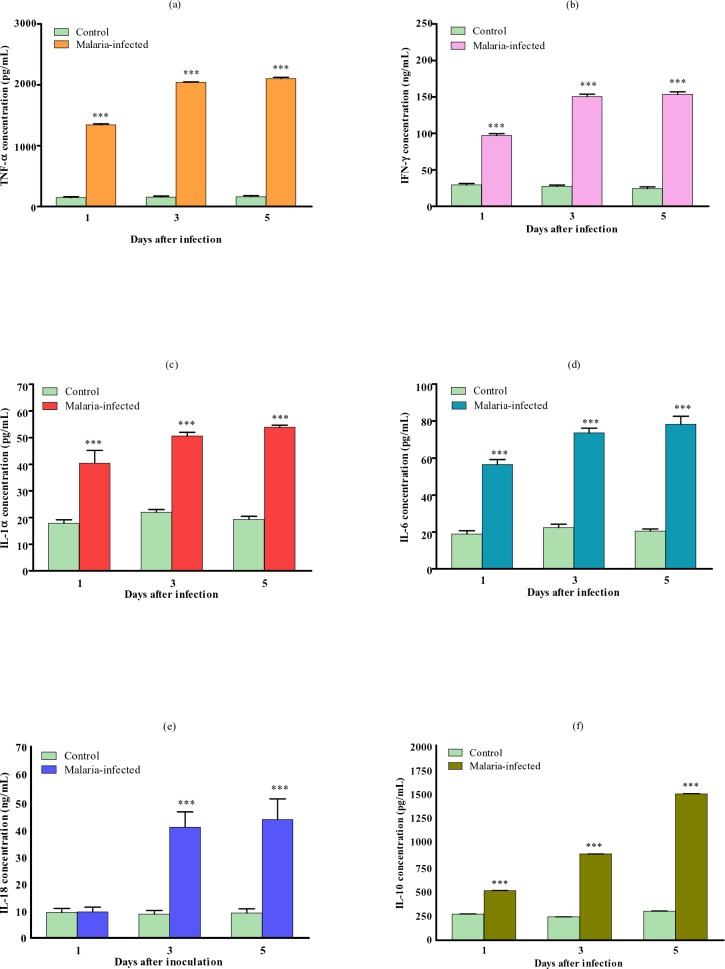
Plasma concentrations of a) TNFα, b) IFNγ, c) IL-1, d) IL-6, e) IL-18 and f) IL-10 of control and malaria-infected mice. Results are mean ± s.e.m of N = 8, ***P<0.0005

In this malarial model, all the pro-inflammatory cytokines, TNFα, IFNγ, IL-1, IL-6 and IL-18 and the anti-inflammatory cytokine IL-10, were significantly elevated in the plasma throughout the infection with the highest level recorded during the late critical stage (Fig. 6).

Relatively high levels of TNFα, IFNγ, IL-1, IL-6 were recorded at the early stage of infection and persisted throughout. IL-10 increment is more gradual with low levels recorded at the start of infection and then gradually increased towards the later stages of infection. IL-18 was only significantly elevated during the middle and late phase of the infection.

### Histopathological findings

**Fig. 7:**
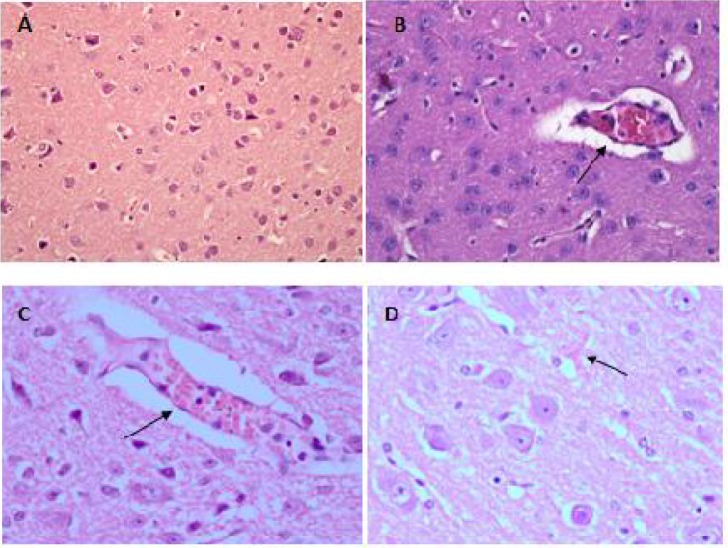
Haematoxylin-Eosin staining of brain tissue showed sequestration of PRBCs in the cerebral microvessels (B) & (C) as compared to the normal uninfected mice (A). Petechial haemorrhages were also observed in the cerebral tissue (D). Specimens were examined under light microscopy at 400x magnification

**Fig. 8:**
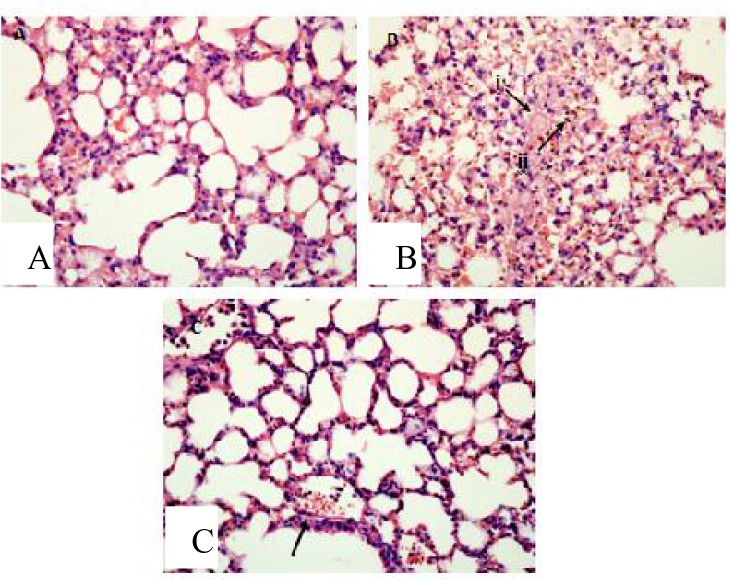
Hyaline membrane formation due to alveolar haemorrhage (B (i)) was seen in the lung tissue of malaria-infected mice as compared to the normal uninfected animal (A). Accumulation of malarial pigment, haemozoin, were also observed (B (ii)), and the alveolar was congested with macrophages engulfing pigments (C). Viewed under light microscopy at 400x

**Fig. 9:**
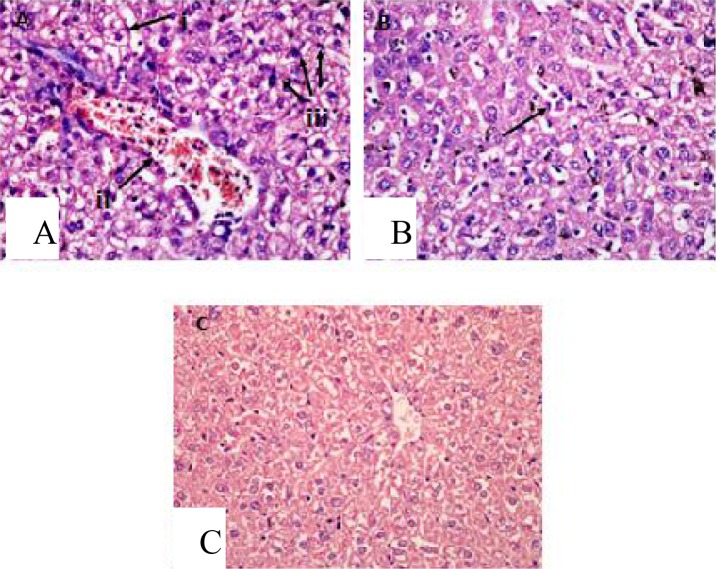
In the liver of malaria-infected mice, hyperplasia and hypertrophy of kupffer cells (A(iii)) with vacuolar degeneration and atrophy of hepatocytes (A(i)) were observed as compared to uninfected mice (C). Microvascular congestion with PRBCs also occurred (A(ii)) leading to congestion of the blood vessels in the liver. Hyperaemia were observed accompanied with capillaries dilatation (B). Viewed under light microscopy at 400x magnification

**Fig. 10:**
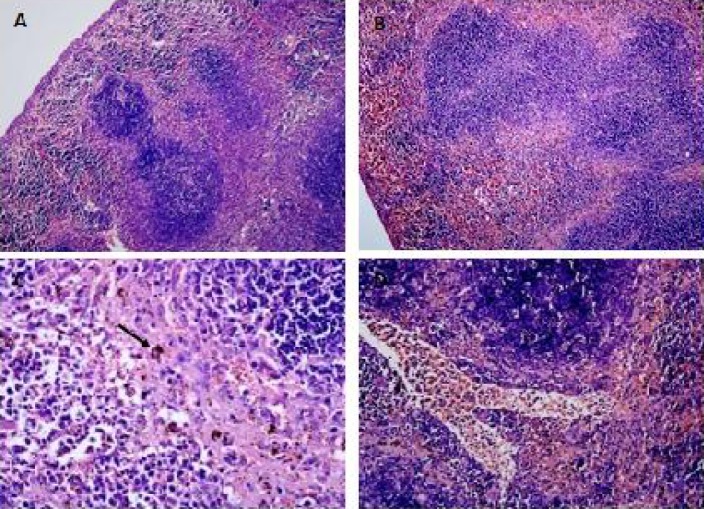
In the spleen of mice infected with malaria, the red and white pulp elements were enlarged accompanied by the lost of typical structure of the germinal centre (B), which is comparable to the normal uninfected mice (A). Accumulation of malarial pigments, haemozoin, was obvious in the pulp histiocytes and sinusoidal lining cells (C). Microvascular sequestrations of PRBCs were also present (D). Viewed under light microscopy at 100x (A & B) and 400x (C & D) magnification

**Fig. 11:**
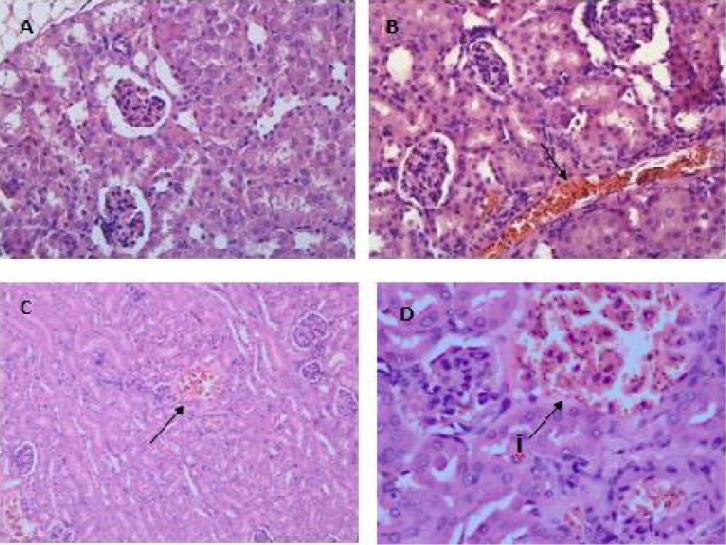
The medulla tissues of the kidney from infected mice were presented with widespread sequestration of PRBCs in the microvascular and interstitium (B), compared to the normal uninfected mouse (A). Haemorrhages (C, D(i)) and congestion (D(i)) of the cortex with PRBCs were also observed. Viewed under light microscopy at 400x (A,B,D) and 200x (C) magnification

Histopathological findings showed that sequestrations of PRBCs in the microvasculature occurred in the brain (Fig. 7B & C), lungs (Fig. 8C), liver (Fig. 9Aii), spleen (Fig. 10D) and kidney (Fig. 11B & D).

Apart from the sequestration of PRBCs in the microvasculature, some other important features of severe malaria were also observed in all the organs. In the brain, mild petechial haemorrhages were observed (Fig. 7D). The lungs from infected mice showed features of hyaline membrane formation which may suggest haemorrhage (Fig. 8B(i)).

The alveolar spaces of the lung were filled with exudates in some areas which may clearly indicates pulmonary edema (Fig. 8C). Haemozoin accumulation occurred in the parenchymals of the lungs (Fig. 8B(ii)) and macrophages engulfing the pigments (Fig. 8C) were observed. In the liver of malarial mice, there were hyperplasia and hypertrophy of the kupffer cells (Fig. 9B). The hepatocytes showed vacuolar degeneration and focal atrophy (Fig. 9C (i)). Hyperaemia accompanied with capillaries dilatation was also clearly observed in the liver (Fig. 9E).

The spleen of malarial mice showed enlargement of the red and white pulps accompanied by the lost of typical structure of the germinal centre (Fig. 10B). Malaria pigments were widely seen in the pulp histiocytes and sinusoidal lining cells (Fig. 10C) and also in the spleen microvasculature (Fig. 10D). The kidney tissues were presented with abundant of PRBC and haemozoin in the microvasculature and interstitium (Fig. 11B). Hyaline cast and vacuolation in the tubules were occasionally observed. Congestions and haemorrhages were seen in the medulla (Fig. 11B) and cortex (Fig. 11C & 11D) of the kidney of infected mice.

## Discussion

Approaches to understanding disease pathogenesis have involved experimental infections in animal model and *in vitro* modelling of the pathogenic processes. In the study of CM, both the experimental animal and *in vitro* models have been central to our understanding of the events leading to cerebral pathology. CM is a major lethal complication of *P. falciparum* infection in human which involved an extremely complex multi-process and multi-system disorder presenting a wide range of clinical features. It is characterized by a sequestration of PRBC as the main important feature, particularly in the deep cerebral microvasculature and by the increased levels of pro-inflammatory cytokines. Prospective studies on the relationship between the immunological events and the neurological dysfunction and also other pathophysiological parameters in human are clearly unethical. Therefore, experimental animal models are necessary to unscramble the fatal pathogenic processes.

Data obtained in this study substantiated that ICR mice are very susceptible to *P. berghei* ANKA infection. Rapid onset of malaria occurred as early as on day 1 following inoculation with the parasite. A high degree of parasitaemia with ultimate death suggests the severe degree of infection in this model. Death is also the ultimate complication of many *falciparum* cases in human. Massive lost in the total number of normal RBC in this model showed a characteristic sign of severe anaemia which is one the major clinical manifestation of severe malaria in human. The pathogenenesis of severe anaemia during malaria infection is complex and involves multiple processes relating to both the destruction and decreased production of erythrocytes (9). During *falciparum* infection, reticulocyte levels are low (10), indicating the suppression of erythropoietin synthesis. Increased RBCs destruction also occurs through the rupture of PRBCs and the phagocytosis of the parasitized and unparasitized RBCs by the hyperactive macrophages in the reticuloendothelial system (10, 11). Severe anaemia has been identified as one of the main mechanisms of severe morbidity and mortality in *falciparum* infection since it can lead to profound hypoxia and congestive cardiac failure (12, 13).

In this study, we measured the body weights of the infected mice to monitor the effects of infection on factors such as food and water intake, metabolism and gut function. The decrease in body weight of malarial mice was clearly evident from the third day of infection, and presumably due in part to the decrease in food intake. Decrease in body weight may also be the consequences of disturbed metabolic function and hypoglycaemia that has been reported to be associated with malaria infection (2). Records on body temperature during the study showed that the mice developed hypothermia upon infection with the parasites. Fever is one of the dramatic manifestations of human malaria. In contrast, this model of malaria was associated with hypothermia rather than pyrexia. The infected mice developed profound hypothermia with colonic temperature falling by as much as 9 °C. The prolonged development of severe hypothermia in mice can be attributed to the general debilitating effects of malaria on the host, which results in the lost of body heat and eventual death. The absence of pyrogenic response in this model is due to the fact that small animals like mice have a large surface area-to-body mass ratio, which resulted in a higher degree of heat loss and prevents the development of fever caused by pyrogenic agents (14). Decreased food intake, indicated by decreased body weight, may also have contributed towards hypothermia. Changes in the physical appearances of vital organs in the body are common during malaria infection. Splenomegaly and hepatomegaly are among the common phenomena (15, 16). Both organs are congested and swollen from the accumulation of the malarial pigment, haemozoin, which led to discolouration. In kidney, congestion can occur in the cortex and medulla during *falciparum* infection (17). Paleness of the brain may be resulted from the severe anemia. Piloerection, one of the physical signs of illness observed in this model, may be related to the hypothermic state during the infection as the homeostatic mechanism is adapting to the heat loss. The passage of dark urine in this model may be equalized with the blackwater fever normally occur in *falciparum* malaria (18, 19).

Inflammatory cytokines have long been implicated in severe malaria. In this model of malaria, all the inflammatory cytokines (TNFα, IFNγ, IL-1, IL-6, IL-18, IL-10) were found to be significantly elevated in the systemic circulation. Similar observations in the elevation of these cytokines levels have been made in human malaria. Findings from the previous studies have revealed the possible role played by each cytokines in the pathogenesis of malaria. Plasma levels of TNFα studied in patients with severe *falciparum* infection showed a significant elevation of the cytokine which often correlates with the cerebral pathology (20, 21, 22). TNFα was thought to be the crucial final mediator of inflammation in CM, but its production is regulated by other pro-inflammatory cytokines including IFNγ and IL-12. IFNγ has been associated with both the pathogenesis and protection against human malaria (23, 24). For IL-6, even though the level is elevated during CM, but it is not thought to be directly involved in the pathogenesis of the disease (25). IL-10 is an immunoregulatory cytokines and has been associated with the amelioration of the pathology and death in murine malaria (26). In children with severe malaria, elevated levels of IL-10 have been reported to play protective role (27, 28). IL-18 is quite a newly identified cytokines among the six reported in this study, but its involvement in malaria infection have been documented in a few studies. IL-18 was found to be highly elevated in this model of malaria, especially during the late critical stages of the infection. Prior study in human have revealed a similar finding in which elevated IL-18 levels were observed in patients with *falciparum* malaria and the levels were significantly higher in severe group as compared to the uncomplicated group (29), with positive correlation observed between IL-18 and the extent of parasitaemia.

Since malaria causes multi-organs dysfunction, we carried out a histopathological study on five major organs known to be affected during malaria infection i.e., the brain, liver, spleen, lungs and kidneys. In human, sequestration of PRBCs in vital organs of the body, particularly in the brain, heart, lungs and liver is a major feature of severe malaria due to infection with *P. falciparum*. Sequestration of PRBC within the brain is the major histopathological feature of human CM. It was suggested that CM is a consequence of the adherence of PRBC to the cerebral microvascular endothelium, leading to vascular obstruction and cerebral hypoxia (5). Sequestrations of PRBC in the microvasculature of all the major organs were also observed in this model of malaria. A few other features mimicking those seen during CM were also observed. In the lungs, hyaline membrane formations suggesting hemorrhages occurred which may lead to ineffective gas exchange through the alveolar membrane. Pulmonary edema was also present which is an important feature of severe malaria and the most dangerous complication of *falciparum* infection (30, 31). In the liver, macrophages engulfed the PRBCs causing lysis of the blood cells leaving behind the haemozoin deposited in the parenchymals. The same histopathology-ical features were observed in the liver during *falciparum* infection in human (32). Splenomegaly observed on the spleen of infected mice in this model also mimic the conditions of *falciparum* infection in human, in which, splenomegaly is a common phenomenon with the spleen becomes congested with malaria haemozoin caused by an increase of the pulp element rendering trabeculae and follicles indistinct (16). Histopathological features of the kidney tissue in this model like hyaline cast and vacuolation in the tubules and also congestions and haemorrhages of the kidney's medulla and cortex, are also typical histopathological features of *falciparum* infection in human (17).

## Conclusions

We have demonstrated in this study that *P. berghei* ANKA infection in ICR mice can reproduce many of the important clinical features of human CM. Therefore, this model can be used to further advance our knowledge and understandings of the pathogenesis of CM and can also be utilized as the first step in the screening of potential new compounds or vaccine candidates to treat severe malaria like CM.
